# Sex differences in metabolic syndrome among U.S. adolescents, NHANES 1999–2020

**DOI:** 10.1186/s13098-025-02052-5

**Published:** 2025-12-11

**Authors:** Danwei Zhang, Xiaoyan Huang, Zihan Chen, Yinan Liu

**Affiliations:** 1 Department of Cardiac Surgery, Fujian Children’s Hospital (Fujian Branch of Shanghai Children’s Medical Center), College of Clinical Medicine for Obstetrics & Gynecology and Pediatrics, Fujian Medical University, No.966 Hengyu Road, Jinan District, Fuzhou, 350014 Fujian China; 2 Department of Endocrinology, Quanzhou First Hospital, Fujian Medical University, Fujian 362000 Quanzhou, China

**Keywords:** Metabolic syndrome (MS), Adolescents, Sex differences, Metabolic phenotype of obesity, National health and nutrition examination survey (NHANES)

## Abstract

**Background:**

Metabolic syndrome (MS) poses a growing threat to adolescent health, yet its sex-specific patterns remain inadequately characterized. This study aimed to investigate sex differences in MS prevalence, risk factor clustering, and metabolic phenotypes among U.S. adolescents.

**Methods:**

We analyzed cross-sectional data from 6,989 adolescents aged 12–19 years, representing 30.97 million U.S. adolescents, from the National Health and Nutrition Examination Survey (1999–2020). MS was defined based on abdominal obesity, elevated blood pressure, hyperglycemia, and dyslipidemia, using weighted analyses to account for complex survey design.

**Results:**

The overall MS prevalence was 5.1%, with a significantly higher prevalence in males than in females (6.1% vs. 4.1%, *P* = 0.017). Critically, no significant temporal trends in MS prevalence were observed over the 20-year period for either sex. Males accumulated more metabolic risk factors than females, showing higher rates of elevated BP (5.9% vs. 0.9%), fasting glucose (22.7% vs. 10.4%), and triglycerides (8.8% vs. 6.4%), whereas, females had higher prevalences of abdominal obesity (25.9% vs. 12.6%) and low HDL-C (22.8% vs. 18.8%). Moreover, metabolically unhealthy obesity was more common in males (13.7% vs. 10.0%). Subgroup analyses showed significant sex disparities persisted among ages 12–15, obese, non-Hispanic White, and high-income subgroups. Sensitivity analyses using alternative definitions, including waist-to-height ratio for abdominal obesity, robustly confirmed the male predominance in MS prevalence.

**Conclusion:**

Male U.S. adolescents exhibited a higher MS prevalence and a more unfavorable aggregation of metabolic risk factors than females. The stability of MS prevalence over the two-decade period highlighted the need for sex-specific and developmentally targeted interventions, especially within identified high-disparity subgroups.

**Supplementary Information:**

The online version contains supplementary material available at 10.1186/s13098-025-02052-5.

## Introduction

Metabolic syndrome (MS), a cluster of cardiometabolic risk factors, is a critical precursor to diabetes and cardiovascular diseases [1]. A systematic review indicated that approximately 5% of adolescents globally had MS, with the prevalence varying across countries and regions [2]. Halting the rise in adolescent cardiometabolic risk factors is crucial to mitigate the global burden of cardiovascular disease.

However, current research predominantly focuses on adults, neglecting the vital transitional phase of adolescence. This period profoundly impacts long-term physical and mental health [3]. Moreover, inconsistent diagnostic criteria for adolescent MS hinder prevalence estimation and valid comparisons [4]. 

Existing studies have insufficiently explored sex differences in MS. This developmental window features sex-specific biological changes: differences in pubertal sex hormones are suggested to significantly contribute to sexual dimorphism in insulin sensitivity and adiposity distribution [5, 6]. Beyond sex-based biological differences, modifiable lifestyle and environmental factors significantly impact MS. For example, diets high in sugar and saturated fats exacerbate insulin resistance; [7,8] physical activity, as a foundation for energy expenditure and physiological function, can modulate metabolic health; [9] furthermore, broader environmental contexts can shape or constrain health behaviors, thereby modulating biological risk. Sex differences in these factors among adolescents may further amplify biological disparities in MS risk [10, 11]. Emerging evidence of greater metabolic impairment in adolescent males underscores the need for sex-stratified analyses to guide targeted interventions [12]. 

This study examined sex-specific patterns in MS using National Health and Nutrition Examination Survey (NHANES) data. We quantified MS prevalence overall and across demographic subgroups, and characterized the distribution of its component risk factors and associated metabolic phenotypes.

## Methods

### Study design

NHANES is a serial, cross-sectional, national survey assessing U.S. population health. The details of NHANES, including detailed survey planning and field operations are described on the official website [13]. The Centers for Disease Control and Prevention (CDC) National Center for Health Statistics (NCHS) Ethics Review Board (ERB) reviewed and approved the NHANES survey protocol, and all participants provided written informed consent [14]. 

The continuous quality assurance (QA) and quality control (QC) of NHANES are core to ensuring high-quality and timely data. Tightly integrated into a two-phase process during data collection: QA (pre-collection) includes equipment calibration and personnel training; QC (in-collection) covers automated software editing, technicians’ performance analysis, and analytical processing [15]. 

### Study population

We analyzed data from adolescents aged 12–19 years across NHANES cycles from1999-2000 to 2017-March 2020 pre-pandemic. The fasting subsample (*n* = 8,307) was included because fasting plasma glucose (FPG) was a key componentfor defining metabolic status. Participants with missing data for key variables, including body mass index (BMI), waist circumference (WC), blood pressure (BP), FPG, triglycerides (TG), or high-density lipoprotein cholesterol (HDL-C), were excluded (final *n* = 6,989; Figure S1). This study was exempted from review by Fujian Children’s Hospital Institutional Review Board as it used publicly de-identified data.

### Data collection and classification

Demographic data were collected through household interviews, includin*g* information on age (12–15 years, 16–19 years), sex, race and ethnicity (non-Hispanic White, non-Hispanic Black, Mexican American, other Hispanic, and other races). Family poverty income ratio (PIR; categorized as low income [< 1.3], middle income [1.3–3.5], and high income [> 3.5]), citizenship, and insurance status were also recorded, as these factors may influence an individual’s access to preventive health services. Smoking status—a recognized risk factor for numerous health conditions, including MS—was obtained from Mobile Examination Center (MEC) assessments. Body examination data, including height, weight, WC, and BP, were collected in the MEC by trained technicians following standardized protocols. BMI was calculated as weight (kg)/height (m)^2^. Age- and sex-specific BMI percentiles were computed using CDC reference data and categorized as: underweight/normal (< 85th), overweight (≥ 85th and < 95th), or obese (≥ 95th). [16] Waist-to-height ratio (WHtR) was calculated as WC (cm)/height (cm). BP was calculated as the mean of all available measurements. Blood samples were collected at the mobile examination centers, stored and sent to central laboratories per standard protocols to assay FPG, TG and HDL-C.

There is currently no universally accepted set of abnormal cutoffs for WC, BP, FPG, TG, and HDL-C for adolescents. In this study, we therefore established abnormality criteria based on a synthesis of multiple reference standards (Table 1, Supplemental methods). Specifically, when the abdominal obesity (AO; assessed by WC) defined by percentiles, the 90th percentile of WC was calculated as sex- and age-specific within study population; while for BP, the age/sex/height-specific 90th percentile was adopted according to international references [17]. 

**Table 1:** Sex differences in metabolic indices under multiple standards

	Overall (*n* = 6989)	Male (*n* = 3644)	Female (*n* = 3345)	*P* value
**Waist circumference (WC)**				
Mean (95% CI), cm	81.78 (81.21, 82.36)	82.16 (81.33, 82.99)	81.38 (80.68, 82.08)	0.140
STD1: <16 years: ≥ 90th percentile or adult cutoff if lower; ≥16 years: >102 cm in males and > 88 cm in females	19.03 (17.5, 20.56)	12.64 (10.9, 14.38)	25.87 (23.49, 28.24)	< 0.001
STD2: ≥102 cm in males and ≥ 88 cm in females	18.66 (17.14, 20.18)	11.92 (10.13, 13.71)	25.87 (23.49, 28.24)	< 0.001
STD3: ≥94 cm in males and ≥ 80 cm in females	31.01 (29.23, 32.8)	19.46 (17.34, 21.59)	43.36 (40.79, 45.94)	< 0.001
**Blood pressure (BP)**				
SBP, mean (95% CI), mmHg	109.36 (108.9, 109.82)	112.22 (111.58, 112.85)	106.3 (105.79, 106.81)	< 0.001
DBP, mean (95% CI), mmHg	60.93 (60.38, 61.47)	59.99 (59.31, 60.67)	61.92 (61.32, 62.52)	< 0.001
STD1: ≥130/85 mmHg	3.5 (2.78, 4.21)	5.92 (4.64, 7.19)	0.91 (0.45, 1.37)	< 0.001
STD2: ≤17 years: ≥90th percentile by age, sex, and height; >17 years: ≥130/85 mmHg	8.55 (7.47, 9.64)	10.2 (8.67, 11.73)	6.79 (5.43, 8.15)	0.001
**Fasting plasma glucose (FPG)**				
Mean (95% CI), mg/dL	94.5 (93.98, 95.01)	96.7 (95.8, 97.59)	92.14 (91.59, 92.7)	< 0.001
STD1: ≥100 mg/dL	16.78 (15.15, 18.4)	22.74 (20.33, 25.15)	10.41 (8.94, 11.87)	< 0.001
STD2: ≥110 mg/dL	2.23 (1.79, 2.68)	2.79 (2.13, 3.45)	1.63 (1.01, 2.25)	0.001
**Triglycerides (TG)**				
Mean (95% CI), mg/dL	81.62 (79.69, 83.54)	83.69 (80.85, 86.53)	79.4 (76.87, 81.93)	0.027
STD1: ≥150 mg/dL	7.66 (6.75, 8.57)	8.85 (7.36, 10.34)	6.39 (5.23, 7.54)	0.013
STD2: ≥110 mg/dL	18.15 (16.51, 19.79)	19.8 (17.69, 21.92)	16.39 (14.27, 18.51)	0.014
**High-density lipoprotein cholesterol (HDL-C)**				
Mean (95% CI), mg/dL	51.85 (51.43, 52.28)	49.73 (49.15, 50.3)	54.13 (53.44, 54.82)	< 0.001
STD1: <16 years: <40 mg/dL; ≥16 years: <40 mg/dL in males and < 50 mg/dL in females	20.72 (19.24, 22.21)	18.77 (16.82, 20.73)	22.81 (20.71, 24.9)	0.005
STD2: ≤40 mg/dL	16.72 (15.36, 18.08)	21.59 (19.53, 23.66)	11.51 (9.97, 13.05)	< 0.001

Data are presented as mean and 95% CI or percentage (%) and 95% CI, adjusted for NHANES fasting sample weight

BP = blood pressure; CI = confidence interval; DBP = diastolic blood pressure; FPG = fasting plasma glucose; HDL-C = high-density lipoprotein cholesterol; NHANES = National Health and Nutrition Examination Survey; SBP = systolic blood pressure; STD = standard; TG = triglycerides; WC = waist circumference

### Definition of metabolic syndrome and metabolically healthy/unhealthy obesity

We applied two classification systems to define MS (details in Supplemental methods). For the 5 metabolic risk factors (AO, high BP, increased FPG, elevated TG, and low HDL-C), the first system adopted the latest joint scientific statement on MS, which declared that having ≥ 3 abnormal findings out of 5 as qualifying a person for MS.^1^ Whereas, the other system taking the International Diabetes Federation (IDF) definition (as sensitivity analysis) stated that AO was an obligatory component, and having ≥ 2 risk factors among the remaining 4 meant MS [18]. We also used WHtR ≥ 0.50 to define AO in the sensitivity analysis [19, 20]. 

We adopted the following definition as primary criterion (Criterion 1) for metabolic risk factors: for participants aged < 16 years, WC ≥ 90th percentile (age- and sex-specific) or males > 102 cm/females > 88 cm (if lower), BP ≥ 130/85 mmHg, FPG ≥ 100 mg/dL, TG ≥ 150 mg/dL and HDL-C < 40 mg/dL; while for ages ≥ 16 years, WC was adjusted to males > 102 cm/females > 88 cm, and HDL-C was modified to males < 40 mg/dL/females < 50 mg/dL. Moreover, another two distinct cutoff criteria were also established, as Criterion 2 took 90th percentile as BP threshold, and Criterion 3 further modified lipid cutoffs (Supplemental methods).

Additionally, metabolic obesity phenotypes—metabolically healthy obesity (MHO) and metabolically unhealthy obesity (MUO)—were classified based on BMI categories and 4 metabolic risk factors (elevated BP, FPG, TG, and reduced HDL-C) to reflect premetabolic syndrome status. Adolescents with obesity and none of these factors were categorized as MHO; all others were considered MUO.

### Statistical analysis

We strictly adhere to the data analysis guidelines published by NHANES for data processing and analysis. All analyses incorporated the NHANES complex survey design and fasting subsample weights to generate nationally representative estimates. Prevalence estimates for categorical variables (e.g., sociodemographic characteristics, MS, MHO and MUO, individual MS components) are presented as weighted proportions with their 95% confidence interval (CI). These estimates were compared between sexes using the Chi-squared (χ²) test, which is appropriate for testing associations between two categorical variables. For continuous variables (e.g., age and metabolic measurements), we present weighted means with 95% CI. Differences between sexes were assessed using t-tests, which is suitable for comparing the means of a continuous variable across two independent groups.

Temporal trends in the prevalence of binary outcomes (e.g., the presence of MS) across the survey cycle were assessed via logistic regression models, treating the survey cycle as a continuous variable. To evaluate whether observed temporal trends differed significantly between males and females, we introduced a multiplicative interaction term between the survey cycle and sex group into the logistic regression model. A statistically significant interaction term would indicate that the trend over time is not parallel between the sexes. The same analytical approach (logistic regression with an interaction term) was applied to assess age-related trends and potential effect modifications by age group.

To explore the potential heterogeneity of effects, subgroup analyses were conducted, stratifying the study population by key categorical variables: age groups, BMI categories, race/ethnicity, and socioeconomic status.

All analyses were performed with R (version 4.2.3; R Core Team, 2023). A two-sided *P* < 0.05 was considered statistically significant. To ensure the reproducibility of our findings, an independent researcher replicated the entire analytical process. The statistical analyses were conducted from November 2024 to May 2025.

## Results

### Study population

The study included 6,989 adolescents with complete interview information, representing 30,974,299 U.S. adolescents. The mean age of the study population was 15.5 years, and 51.7% were males. There were no significant sex differences in sociodemographic characteristics (Table 2), including age, race/ethnicity, PIR, citizenship, and health insurance status. For health status, both sexes had similar BMIs, but females showed a higher proportion of individuals with a WHtR ≥ 0.5 (males vs. females: 30.7% vs. 41.6%, *P* < 0.001), while males had a higher smoking proportion (14.9% vs. 11.3%, *P* = 0.01).

**Table 2:** Population characteristics of adolescents aged 12–19 years in NHANES

	Overall (*n* = 6989)	Male (*n* = 3644)	Female (*n* = 3345)	*P* value
**Weighted number of population**	30,974,299	16,003,274	14,971,025	
**Age**,** mean (95% CI)**,** years**	15.49 (15.41, 15.57)	15.53 (15.42, 15.64)	15.45 (15.32, 15.57)	0.338
**Age group**,** years**				0.999
12–15	49.85 (48.13, 51.57)	49.85 (47.42, 52.28)	49.85 (47.26, 52.45)	
16–19	50.15 (48.43, 51.87)	50.15 (47.72, 52.58)	50.15 (47.55, 52.74)	
**Survey cycle**				1.000
1999–2000	8.23 (6.95, 9.51)	8.43 (6.86, 10)	8.02 (6.69, 9.35)	
2001–2002	10.41 (8.91, 11.92)	10.07 (8.28, 11.86)	10.78 (8.96, 12.6)	
2003–2004	9.49 (8.15, 10.82)	9.41 (7.72, 11.1)	9.57 (7.98, 11.16)	
2005–2006	9.91 (8.47, 11.36)	9.83 (7.91, 11.75)	10 (8.4, 11.6)	
2007–2008	9.8 (8.43, 11.17)	9.83 (8.07, 11.59)	9.77 (7.76, 11.78)	
2009–2010	9.28 (7.88, 10.69)	9.37 (8.12, 10.63)	9.19 (6.9, 11.47)	
2011–2012	9.54 (7.91, 11.16)	9.59 (7.64, 11.53)	9.49 (7.57, 11.4)	
2013–2014	9.56 (8.01, 11.12)	9.56 (7.53, 11.59)	9.57 (8.04, 11.1)	
2015–2016	9.57 (8.08, 11.06)	9.64 (7.93, 11.35)	9.49 (7.86, 11.13)	
2017–2020	14.2 (12.09, 16.3)	14.27 (12.07, 16.47)	14.12 (10.87, 17.36)	
**Race/ethnicity**				0.650
Non-Hispanic White	58.62 (55.89, 61.35)	58.88 (55.78, 61.98)	58.33 (55.09, 61.58)	
Non-Hispanic Black	14.22 (12.58, 15.86)	13.69 (11.94, 15.43)	14.79 (12.83, 16.76)	
Mexican American	13.16 (11.45, 14.87)	13.19 (11.28, 15.09)	13.12 (11.21, 15.04)	
Other Hispanic	6.21 (5.12, 7.3)	6.06 (4.78, 7.34)	6.37 (5.13, 7.61)	
Other Race	7.79 (6.67, 8.92)	8.18 (6.71, 9.65)	7.38 (6.02, 8.74)	
**Family poverty income ratio**				0.876
< 1.3	28.42 (26.33, 30.51)	27.88 (25.39, 30.37)	29.01 (26.48, 31.53)	
1.3–3.5	34.4 (32.06, 36.75)	34.72 (32.02, 37.43)	34.06 (30.9, 37.22)	
>3.5	30.94 (28.56, 33.32)	31.3 (28.32, 34.29)	30.56 (27.39, 33.74)	
Missing	6.23 (5.26, 7.2)	6.1 (4.97, 7.22)	6.37 (4.89, 7.85)	
**Citizen by birth or naturalization**	80.51 (78.31, 82.71)	79.92 (77.56, 82.29)	81.14 (77.89, 84.38)	0.479
**Have health insurance**	87.2 (85.98, 88.42)	86.86 (85.22, 88.5)	87.57 (85.86, 89.28)	0.615
**WHtR ≥ 0.5**	35.93 (34.11, 37.75)	30.65 (28.33, 32.98)	41.57 (39.05, 44.1)	< 0.001
**BMI categories**				0.639
Underweight and normal weight	64.27 (62.7, 65.83)	64.72 (62.42, 67.02)	63.79 (61.43, 66.14)	
Overweight	15.99 (14.75, 17.24)	15.39 (13.65, 17.13)	16.63 (14.96, 18.31)	
Obese	19.74 (18.26, 21.22)	19.89 (17.65, 22.13)	19.58 (17.55, 21.61)	
**Smoking status**				
Recent smoke	13.16 (11.8, 14.52)	14.9 (13.15, 16.65)	11.3 (9.55, 13.05)	0.010
Anyone smoke in home	15.93 (14.21, 17.65)	16.17 (13.94, 18.41)	15.67 (13.67, 17.67)	0.578

Data are presented as mean and 95% CI or percentage (%) and 95% CI, adjusted for NHANES fasting sample weight

BMI = body mass index; CI = confidence interval; NHANES = National Health and Nutrition Examination Survey; PIR = family poverty income ratio; WHtR = waist-to-height ratio

### Sex differences in metabolic indices

The mean value of WC was similar between males and females, but BP, FPG, TG and HDL-C differed significantly between sexes (Table 1). Based on these, the proportion of elevated WC was higher among females, reaching 25.9%, nearly twice that of males (12.6%, *P* < 0.001). Besides, a relatively higher proportion of abnormalities among females was also found in the case of low HDL-C, which accounted for 22.8%, compared with 18.8% among males (*P* = 0.005). In contrast, abnormally elevated values of BP (males vs. females: 5.9% vs. 0.9%, *P* < 0.001), FPG (22.7% vs. 10.4%, *P* < 0.001), and TG (8.8% vs. 6.4%, *P* = 0.013) were observed in a greater proportion of males than females. Temporal changes in metabolic indices are presented in Figure S2. No significant differences in trends were observed between sexes (all P for interaction > 0.05), and only FPG exhibited a gradual increase over time in both sexes (*P* < 0.001). When stratified by age (Table S1), sex differences emerged in all metabolic indices except TG levels in 12–15-year-olds. Across BMI categories (Table S2), most metabolic parameters showed significant sex-based variations, though no sex differences persisted in TG among individuals with underweight/normal weight or in HDL-C in individuals with obesity.

A BP ≥ 90th percentile (by age, sex, and height) is also commonly used to define elevated BP in adolescents. This increased the prevalence of elevated BP in both sexes while reducing sex-based disparity from 6.5-fold (male vs. female: 5.9% vs. 0.9%) to 1.5-fold (10.2% vs. 6.8%, *P* = 0.001, Table 1). Results under other standards proposed by previous studies also revealed significant sex differences (Table 1). Supplemental materials include analyses stratified by age group (Table S1), BMI group (Table S2), and temporal trends (Figure S2).

### Sex differences in metabolic syndrome

According to the primary MS criterion adopted in this study, the overall prevalence of MS was 5.1% in U.S. adolescents. Specifically, the proportion of males was relatively higher, reaching 6.1%, than that of females, which was 4.1% (*P* = 0.017, Table 3). Around one-fifth of the adolescents with MS had more than three metabolic risk factors. Moreover, nearly 7% of the males exhibited all five metabolic risk factors, but such a situation was rare in females, and significant differences were observed in the number of metabolic risk factors between sexes (*P* = 0.005, Table 3). For MS among both sexes, the first two combinations of metabolic risk factors were AO with high TG and low HDL-C, and AO with high FPG and low HDL-C, which together accounted for 37.0% and 70.0% of MS in males and females, respectively. All combination forms by sex are detailed in Figure S3.

**Table 3:** Sex differences in metabolic syndrome and metabolic phenotype of obesity

	Overall (*n* = 6989)	Male (*n* = 3644)	Female (*n* = 3345)	*P* value
**Metabolic syndrome**	5.13 (4.31, 5.94)	6.11 (4.86, 7.37)	4.07 (3.02, 5.11)	0.017
Number of risk factors				0.005
3	4.11 (3.37, 4.85)	4.84 (3.68, 5.99)	3.33 (2.44, 4.22)	
4	0.8 (0.48, 1.12)	0.86 (0.43, 1.29)	0.73 (0.26, 1.2)	
5	0.22 (0.08, 0.36)	0.42 (0.15, 0.68)	0.01 (−0.01, 0.03)	
Constituent ratio of Top 5 risk factors combinations in MS^a^				
AO, High TG, Low HDL-C	29.9 (22.89, 36.91)	20.37 (13.37, 27.37)	45.22 (33.16, 57.28)	
AO, High FPG, Low HDL-C	19.72 (14.65, 24.78)	16.65 (9.85, 23.44)	24.65 (15.4, 33.91)	
AO, High FPG, High TG, Low HDL-C	10.94 (6.02, 15.86)	9.84 (3.91, 15.78)	12.71 (4.28, 21.13)	
AO, High BP, High FPG	8.02 (3.22, 12.82)	12.17 (4.54, 19.8)	1.36 (−0.59, 3.31)	
High FPG, High TG, Low HDL-C	7.2 (3.11, 11.29)	8.5 (2.86, 14.13)	5.11 (0.02, 10.2)	
**Metabolic phenotype of obesity**				
MHO	7.84 (6.93, 8.74)	6.22 (5.04, 7.4)	9.57 (7.96, 11.18)	0.002
MUO	11.9 (10.77, 13.03)	13.67 (11.85, 15.49)	10.01 (8.64, 11.38)	0.002

Data are presented as percentage (%) and 95% CI, adjusted for NHANES fasting sample weight

AO = abdominal obesity; BP = blood pressure; CI = confidence interval; FPG = fasting plasma glucose; HDL-C = high-density lipoprotein cholesterol; MHO = metabolically healthy obesity; MUO = metabolically unhealthy obesity; NHANES = National Health and Nutrition Examination Survey; TG = triglycerides

^a^ The five risk factor combinations with the highest proportions in the total study population are listed in sequence. Therefore, the combination ranked fifth among females is not shown in this table. It is AO, high BP, high FPG, and low HDL-C, accounting for 4.08% of the total. All the risk factor combinations are shown in Figure S3

**Fig. 1:**
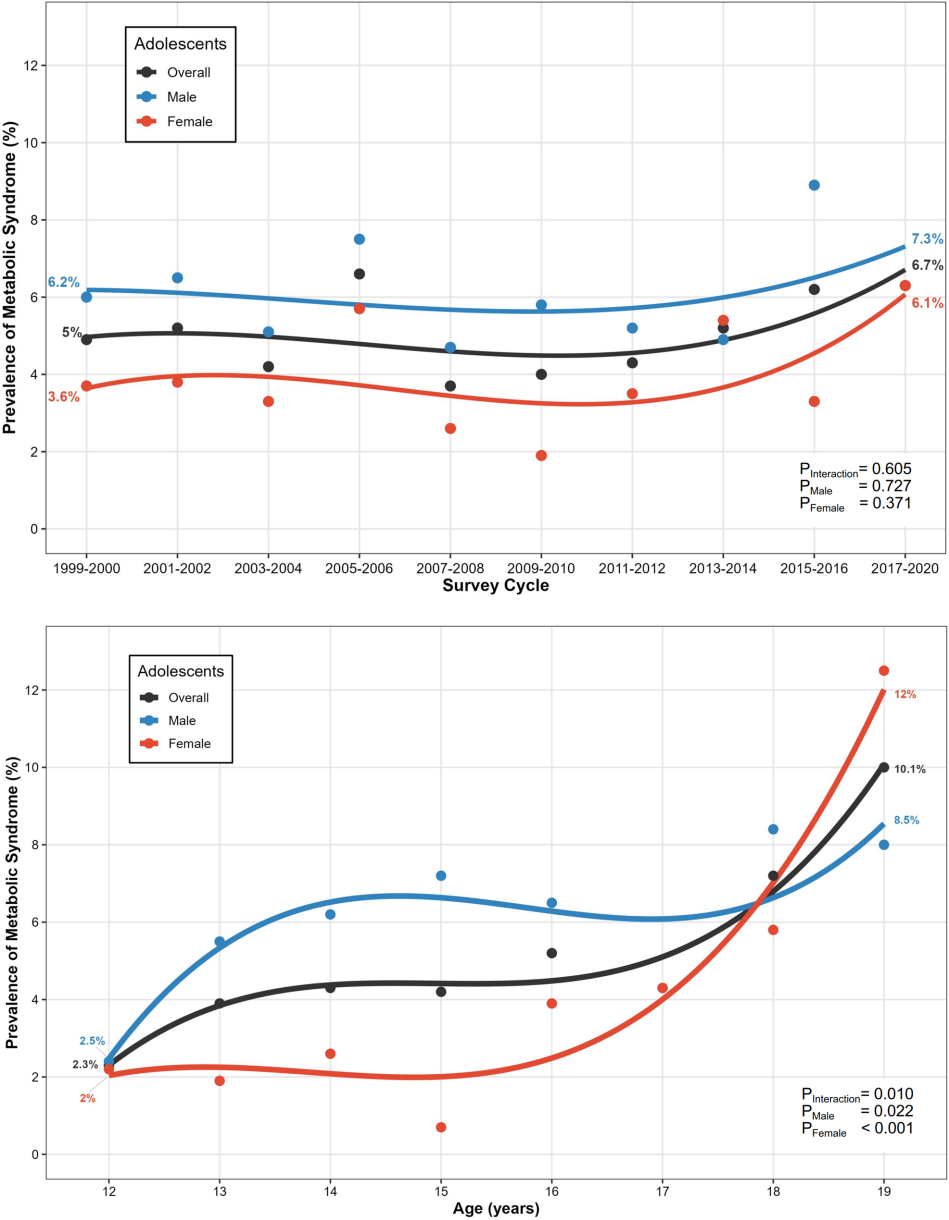
Temporal and Age-Specific Trends in Adolescent Metabolic Syndrome Prevalence. The upper figure illustrates temporal trends (1999–2020) in MS prevalence for the U.S. adolescents aged 12–19 years (black), adolescent males (blue) and females (red), with dots representing survey-period prevalence estimates and curves showing fitted trends; sex-specific temporal trends and differences were statistically tested via logistic regression. Similarly, the lower figure displays age-specific MS prevalence trends for the same groups (with corresponding colors), where dots denote age-stratified prevalence and curves reflect fitted age trends, reporting P values for trend test

**Fig. 2:**
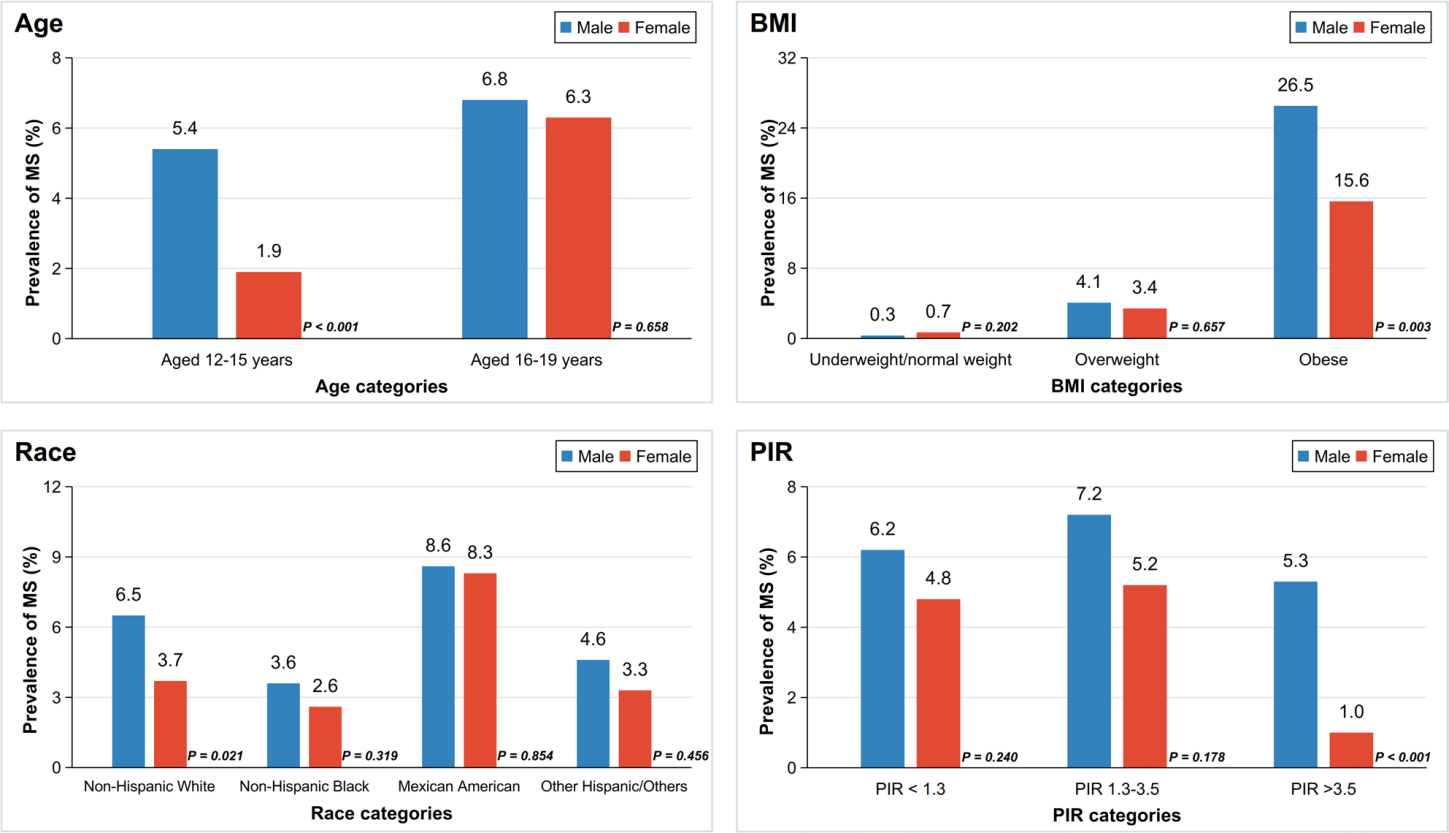
Prevalence of Metabolic Syndrome in Adolescents by Subgroups The bar charts sequentially present sex differences in MS prevalence among US adolescents aged 12–19 years across subgroups stratified by age groups, BMI categories, race/ethnicity, and socioeconomic status; blue bars represent males, red bars represent females, and p values (derived from χ^2^ tests) for sex comparisons are annotated adjacent to the corresponding bars. BMI = body mass index. PIR = family poverty income ratio

No temporal trends were observed in either sex across the study years (*P* > 0.05, Fig. 1). When stratified by age group, males had a significantly greater MS prevalence than females among 12–15-year-olds (5.4% vs. 1.9%, *P* < 0.001) but not among 16–19-year-olds (6.8% vs. 6.3%, *P* = 0.658, Fig. 2). Age-trend analysis (Fig. 1) further showed MS prevalence increased with age in both sexes (*P* < 0.05 for each), particularly in females. Subgroup analyses indicated that only subgroups of obese, Non-Hispanic White, and high income presented sex-difference in MS prevalence (Fig. 2).

When age-, sex-, and height-specific BP thresholds were used (Criterion 2, Table S3), MS prevalence demonstrated modest increases in both males (6.5%) and females (4.8%). Although no significant sex difference emerged for MS (*P* = 0.055), distinct sex disparities persisted in the number of risk factors clustering (*P* = 0.039). When implementing another dyslipidemia criterion (TG ≥ 110 mg/dL and HDL-C ≤ 40 mg/dL in Criterion 3, Table S3), the MS prevalence demonstrated marked elevation (males vs. females: 9.0% vs. 5.6%), with sex differences observed in the MS prevalence (*P* < 0.001) and cumulative risk factor clustering (*P* < 0.001). Temporal and age-specific trends, and prevalence patterns of subgroups under different MS criteria are detailed in Figures S4 and S5.

### Sex differences in the metabolic obesity phenotype, MHO and MUO

On the basis of the definitions of MHO and MUO in the method section, males had a lower proportion of MHO than females (Criterion 1: 6.2% vs. 9.6%, *P* = 0.002). In contrast, the proportion of MUO was significantly higher in males than in females (13.7% vs. 6.2%, *P* = 0.002, Table 3). Similar results were obtained when risk factors taking alternative definitions (Table S4).

### Sensitivity analysis

Some studies considered AO a mandatory condition for MS. In the sensitivity analysis (Table S3), the prevalence of MS in adolescents under this definition was calculated. Results showed that the MS prevalence in males was higher than that in females, but the difference was insignificant (Criterion 1: 5.1% vs. 3.9%, *P* = 0.127). However, the count of risk factor clustering was significantly higher in males than in females (*P* < 0.001, Table S3). Moreover, among adolescents with AO, the proportion of MS differed between sexes (males: 40.2% vs. females: 14.9%, *P* < 0.001).

When a WHtR ≥ 0.5 was used as the AO threshold, the MS prevalence increased to 8.2% in males and 4.7% in females (*P* < 0.001, Criterion 1 for other metabolic risk factors). The between-sex differences were persistent across different criteria (Table S5).

## Discussion

This national study revealed a persistently higher prevalence of MS and greater accumulation of metabolic risk factors among male versus female U.S. adolescents (1999–2020), despite overall stable MS trends. Moreover, the study comprehensively examined sex differences in metabolic risk factors and metabolic obesity phenotypes. These findings underscore the critical need for sex-stratified approaches to adolescent metabolic health intervention.

### Sex-specific burden and mechanisms in MS and its components

The predominant finding demonstrated significantly higher MS prevalence and greater cumulative risk factor loads in male than female adolescents. According to different criteria, the prevalence of MS in males ranged from 5.1% to 9.0%, and in females from 3.9% to 5.6% (when AO was defined by WC, Table S3), with consistent significant between-sex difference. Prior studies also observed this phenomenon, [21–24] but specific values and sex differences varied across studies due to population selection, definitions, and use of earlier data (see Table S6 for details). We found no significant change in MS prevalence over 20 years, but updated knowledge on sex-specific MS burden.

Our findings indicated that AO was the most prevalent component in MS in both sexes. Notably, when AO (defined by WC) was mandated as an essential diagnostic criterion, the observed sex difference attenuated (Table S3). This requires cautious interpretation: Due to sex-specific fat distribution patterns [25]—where females typically exhibit greater subcutaneous adipose tissue, probably resulting in elevating puberty WC measurements, whereas males accumulate higher-risk visceral adiposity even below conventional WC thresholds—using AO as a required diagnostic criterion likely underestimates the pathogenic adiposity in adolescent males while potentially overestimating it in females. Supporting this, when AO was defined by WHtR, which is considered to be more capable of reflecting the accumulation of visceral fat, MS prevalence remained consistently higher in males (Table S5).

Component-level analyses revealed pronounced sex-specific patterns: males showed significantly higher percentages of elevated BP, hyperglycemia, and hypertriglyceridemia, while females exhibited higher AO prevalence and reduced HDL-C levels. These disparities originate in puberty through sexual dimorphism in body composition and hormonal regulation, together with dynamic physiological changes during adolescence. Hormonally driven sexual divergence leads to males developing greater muscle mass but higher visceral adiposity, whereas females accumulate more subcutaneous fat (potentially explaining higher AO prevalence, as previously discussed) and exhibit elevated baseline leptin levels [26, 27]. Sex hormones such as estrogen and testosterone critically regulate insulin sensitivity and resistance, with evidence suggesting that estrogen confers protective effects [27]. Different levels of other hormones (e.g., growth hormone and thyroid hormone) further modulate metabolic physiology [26]. Consequently, males demonstrate higher prevalence of impaired fasting glucose, characterized predominantly by raised hepatic glucose output and early insulin secretion defects; [28] while females possess more efficient fat storage mechanisms that improve lipid metabolism [29]—explaining our observed sex differences in FPG, TG and HDL-C profiles. Notably, in females aged ≥ 16, HDL-C thresholds significantly impacted abnormal HDL-C prevalence (36.4% vs. 11.5% for < 50 mg/dL vs. ≤ 40 mg/dL) and reversed the sex disparity in abnormal HDL-C. Adopting stricter female-specific HDL-C criterion is probably more justified given higher baseline levels in females and sex-dependent associations with CVD risk [30]. Adolescent females exhibit lower SBP than males, primarily attributable to reduced stroke volume during physical stress and lower peripheral resistance during mental stress, modulated by body composition and insulin resistance [31]. A study reported that sex differences in SBP emerge primarily during prepuberty, with males showing higher values and steeper adolescent increases; further divergence occurs within 5 years postpuberty [32]. Cardiometabolic risk factors share pathophysiology and respond to lifestyle interventions, emphasizing the need to target adolescents with clustered risks [33]. In general, puberty marks a critical divergence point for sex-specific metabolic trajectories, establishing adolescence as a key phase for sex-tailored preventive strategies.

### Clinical implications of subgroup heterogeneity in MS

Subgroup analyses identified critical patterns for targeted intervention. Regarding age subgroups, the male disadvantage in MS prevalence was most pronounced at 12–15 years; Females showed a steeper rise in MS incidence during 16–19 years, narrowing the sex gap. This supports earlier risk factor intervention in males and intensified monitoring of metabolic parameter changes (such as WC and HDL-C) in females during late adolescence.

BMI subgroup analysis underscored the impact of obesity, as it dramatically increased MS risk (>20% prevalence vs. < 1% in normal weight) as well as markedly widened sex disparities (26.5% in males vs. 15.6% in females). This aligns with prior research that MS prevalence is high in obese adolescents and rises with worsening obesity severity [34]. Despite comparable obesity prevalences, males had significantly higher proportions of MUO than females. We incorporated the concept of MHO/MUO to advance understanding of early metabolic health stages and facilitate early identification of high-risk individuals. Consequently, males carry higher risks of potential metabolic abnormalities, necessitating more timely intervention. Furthermore, given the instability of MHO and its high likelihood of progressing to MUO, monitoring and preventive strategies remain essential [35]. 

Analysis of racial/ethnic subgroups found that Mexican American adolescents showed the highest MS burden in both males and females, without significant sex disparity, warranting comprehensive population-specific interventions, potentially encompassing genetic, dietary, sociocultural, and other prevalent aspects. Significant sex differences existed exclusively in the Non-Hispanic Whites, highlighting unmet needs for sex-focused mechanistic research in this group.

Finally, a significant sex disparity (male predominance in MS) was observed specifically within the high-income group (PIR >3.5). This pattern suggests that the protective effect of high socioeconomic status may be more pronounced in female adolescents. We hypothesize that potentially due to a stronger buffering effect against social stress, gendered differences in health behaviors, or a greater propensity to utilize resources for health promotion for adolescent females from a high-resource environment [36–38]. These potential mediators warrant future investigation to explain this sex-specific protective effect.

### Advantages and limitations of the study

Our study used nationally representative serial data spanning two decades and comprehensively analyzed sex differences in MS, detailed component and phenotype using multiple definitions among U.S. adolescents, with multidimensional subgroup stratification. These not only ensured the temporal comparability of our findings but also enhanced the comparability with other relevant studies. In addition, we provided a foundation for developing sex-specific and subgroup-targeted strategies to mitigate metabolic risk during the critical developmental window of adolescence.

This study has several limitations inherent to its design and scope. First, the cross-sectional nature precluded tracking dynamic metabolic changes during adolescent development. Second, despite efforts to standardize methodologies, residual methodological variations across the 20-year data collection period remain possible. Third, large-scale feasibility constraints limited collected indicators to routine biomarkers, and some were not assessed in younger children, precluding analysis of this group. Finally, we mainly synthesized evidence to define MS, while establishing universally applicable adolescent diagnostic criteria—particularly for optimal AO cutoffs [39]—requires future prospective validation across diverse populations.

## Conclusion

Male adolescents presented a significantly greater MS prevalence and more aggregated risk factors than female adolescents. The MS prevalence remained stable during the study period, suggesting that more vigorous interventions targeting vulnerable metabolic components or key subgroups are needed.

## Supplementary Information

Supplementary Material 1

## Data Availability

The datasets analyzed during the current study are available in the National Health and Nutrition Examination Survey (NHANES), a public use repository at https://wwwn.cdc.gov/nchs/nhanes/default.aspx.

## References

[1] Alberti KGMM, Eckel RH, Grundy SM, et al. Harmonizing the metabolic syndrome: a joint interim statement of the International Diabetes Federation Task Force on Epidemiology and Prevention; National Heart, Lung, and Blood Institute; American Heart Association; World Heart Federation; International Atherosclerosis Society; and International Association for the Study of Obesity. Circulation. 2009;120(16):1640–5. 10.1161/CIRCULATIONAHA.109.192644. 10.1161/CIRCULATIONAHA.109.192644 19805654

[2] Noubiap JJ, Nansseu JR, Lontchi-Yimagou E, et al. Global, regional, and country estimates of metabolic syndrome burden in children and adolescents in 2020: a systematic review and modelling analysis. The Lancet Child & Adolescent Health. 2022;6(3):158–70. 10.1016/S2352-4642(21)00374-6. 35051409 10.1016/S2352-4642(21)00374-6

[3] Sawyer SM, Afifi RA, Bearinger LH, et al. Adolescence: a foundation for future health. Lancet. 2012;379(9826):1630–40. 10.1016/S0140-6736(12)60072-5. 22538178 10.1016/S0140-6736(12)60072-5

[4] Zimmet P, Alberti KGM, Kaufman F, et al. The metabolic syndrome in children and adolescents - an IDF consensus report. Pediatr Diabetes. 2007;8(5):299–306. 17850473 10.1111/j.1399-5448.2007.00271.x

[5] Tramunt B, Smati S, Grandgeorge N, et al. Sex differences in metabolic regulation and diabetes susceptibility. Diabetologia. 2020;63(3):453–61. 10.1007/s00125-019-05040-3. 31754750 10.1007/s00125-019-05040-3 PMC6997275

[6] Mauvais-Jarvis F. Sex differences in energy metabolism: natural selection, mechanisms and consequences. Nat Rev Nephrol. 2024;20(1):56–69. 10.1038/s41581-023-00781-2. 37923858 10.1038/s41581-023-00781-2

[7] Huang Y, Chen Z, Chen B, et al. Dietary sugar consumption and health: umbrella review. BMJ. 2023;381:e071609. 10.1136/bmj-2022-071609. 37019448 10.1136/bmj-2022-071609 PMC10074550

[8] Sakamoto K, Butera MA, Zhou C, et al. Overnutrition causes insulin resistance and metabolic disorder through increased sympathetic nervous system activity. Cell Metab. 2024;37(1). 10.1016/j.cmet.2024.09.012. 10.1016/j.cmet.2024.09.012 PMC11711004 39437790

[9] Kuramoto K, Liang H, Hong J-H, He C. Exercise-activated hepatic autophagy via the FN1-α5β1 integrin pathway drives metabolic benefits of exercise. Cell Metab. 2023. 10.1016/j.cmet.2023.01.011. 36812915 10.1016/j.cmet.2023.01.011 PMC10079584

[10] Qorbani M, Khashayar P, Rastad H, et al. Association of dietary behaviors, biochemical, and lifestyle factors with metabolic phenotypes of obesity in children and adolescents. Diabetol Metab Syndr. 2020;12(1):108. 10.1186/s13098-020-00617-0. 33372634 10.1186/s13098-020-00617-0 PMC7720466

[11] Lee AM, Gurka MJ, DeBoer MD. Trends in metabolic syndrome severity and lifestyle factors among adolescents. Pediatrics. 2016;137(3):e20153177. 10.1542/peds.2015-3177. 26908664 10.1542/peds.2015-3177 PMC4771130

[12] Perng W, Rifas-Shiman SL, Hivert MF, Chavarro JE, Sordillo J, Oken E. Metabolic trajectories across early adolescence: differences by sex, weight, pubertal status and race/ethnicity. Ann Hum Biol May. 2019;46(3):205–14. 10.1080/03014460.2019.1638967. 10.1080/03014460.2019.1638967 PMC6960375 31264447

[13] Centers for Disease Control and Prevention NCfHS. National Health and Nutrition Examination Survey. Accessed May 05. 2025. https://www.cdc.gov/nchs/nhanes/

[14] Centers for Disease Control and Prevention NCfHS. Ethics Review Board Approval. Updated December 18. 2024. Accessed May 05, 2025. https://www.cdc.gov/nchs/nhanes/about/erb.html

[15] Berman LE, Fisher AL, Ostchega Y, Reed-Gillette DS, Stammerjohn EL. Quality Assurance (QC)/Quality Control (QC) Processes for the National Health and Nutrition Examination Survey (NHANES). *Proc AMIA Symp*. 2001:862.

[16] Centers for Disease Control and Prevention NCfHS. Data file for the Extended CDC BMI-for-age Growth Charts for Children and Adolescents. Updated September 02, 2024. Accessed May 05. 2025. https://www.cdc.gov/growthcharts/extended-bmi-data-files.htm

[17] Xi B, Zong Xn, Kelishadi R, et al. Establishing international blood pressure references among nonoverweight children and adolescents aged 6 to 17 years. Circulation. 2016;133(4):398–408. 10.1161/CIRCULATIONAHA.115.017936. 26671979 10.1161/CIRCULATIONAHA.115.017936 PMC4729639

[18] Alberti KGMM, Zimmet P, Shaw J. The metabolic syndrome—a new worldwide definition. Lancet. 2005;366(9491):1059–62. 16182882 10.1016/S0140-6736(05)67402-8

[19] Ashwell M, Gibson S. A proposal for a primary screening tool: ‘Keep your waist circumference to less than half your height’. BMC Med. 2014;12:207. 10.1186/s12916-014-0207-1. 25377944 10.1186/s12916-014-0207-1 PMC4223160

[20] Zhao M, López-Bermejo A, Caserta CA, et al. Metabolically healthy obesity and high carotid Intima-Media thickness in children and adolescents: international childhood vascular structure evaluation consortium. Diabetes Care. 2019;42(1):119–25. 10.2337/dc18-1536. 30420475 10.2337/dc18-1536

[21] de Ferranti SD, Gauvreau K, Ludwig DS, Neufeld EJ, Newburger JW, Rifai N. Prevalence of the metabolic syndrome in American adolescents: findings from the third National health and nutrition examination survey. Circulation. 2004;110(16):2494–7. 15477412 10.1161/01.CIR.0000145117.40114.C7

[22] Liu J, Ma J, Orekoya O, Vangeepuram N, Liu J. Trends in metabolic syndrome among US youth, from 1999 to 2018. JAMA Pediatr. 2022;176(10):1043–5. 10.1001/jamapediatrics.2022.1850. 35816325 10.1001/jamapediatrics.2022.1850 PMC9274445

[23] Ford ES, Ajani UA, Mokdad AH. The metabolic syndrome and concentrations of C-reactive protein among U.S. youth. Diabetes Care. 2005;28(4):878–81. 15793189 10.2337/diacare.28.4.878

[24] Cook S, Weitzman M, Auinger P, Nguyen M, Dietz WH. Prevalence of a metabolic syndrome phenotype in adolescents: findings from the third National health and nutrition examination Survey, 1988–1994. Arch Pediatr Adolesc Med. 2003;157(8):821–7. 12912790 10.1001/archpedi.157.8.821

[25] Costa DN, Santosa S, Jensen MD. Sex differences in the metabolism of glucose and fatty acids by adipose tissue and skeletal muscle in humans. Physiol Rev. 2025;105(3):897–934. 10.1152/physrev.00008.2024. 39869194 10.1152/physrev.00008.2024 PMC12139471

[26] Veldhuis JD, Roemmich JN, Richmond EJ, et al. Endocrine control of body composition in infancy, childhood, and puberty. Endocr Rev. 2005;26(1):114–46. 15689575 10.1210/er.2003-0038

[27] Ciarambino T, Crispino P, Guarisco G, Giordano M. Gender differences in insulin resistance: new knowledge and perspectives. Curr Issues Mol Biol. 2023;45(10):7845–61. 10.3390/cimb45100496. 37886939 10.3390/cimb45100496 PMC10605445

[28] Unwin N, Shaw J, Zimmet P, Alberti KGMM. Impaired glucose tolerance and impaired fasting glycaemia: the current status on definition and intervention. Diabet Med. 2002;19(9):708–23. 12207806 10.1046/j.1464-5491.2002.00835.x

[29] Gado M, Tsaousidou E, Bornstein SR, Perakakis N. Sex-based differences in insulin resistance. J Endocrinol. 2024. 10.1530/JOE-23-0245. 38265844 10.1530/JOE-23-0245

[30] Ko DT, Alter DA, Guo H, et al. High-density lipoprotein cholesterol and cause-specific mortality in individuals without previous cardiovascular conditions: the CANHEART study. J Am Coll Cardiol. 2016;68(19):2073–83. 10.1016/j.jacc.2016.08.038. 27810046 10.1016/j.jacc.2016.08.038

[31] Syme C, Abrahamowicz M, Leonard GT, et al. Sex differences in blood pressure and its relationship to body composition and metabolism in adolescence. Arch Pediatr Adolesc Med. 2009;163(9):818–25. 10.1001/archpediatrics.2009.92. 19736335 10.1001/archpediatrics.2009.92

[32] O’Neill KN, Bell JA, Davey Smith G, Tilling K, Kearney PM, O’Keeffe LM. Puberty timing and sex-specific trajectories of systolic blood pressure: a prospective cohort study. Hypertension. 2022;79(8):1755–64. 10.1161/HYPERTENSIONAHA.121.18531. 35587023 10.1161/HYPERTENSIONAHA.121.18531 PMC9278704

[33] Magge SN, Goodman E, Armstrong SC. The metabolic syndrome in children and adolescents: shifting the focus to cardiometabolic risk factor clustering. Pediatrics. 2017. 10.1542/peds.2017-1603. 28739653 10.1542/peds.2017-1603

[34] Weiss R, Dziura J, Burgert TS, et al. Obesity and the metabolic syndrome in children and adolescents. N Engl J Med. 2004;350(23):2362–74. 15175438 10.1056/NEJMoa031049

[35] Eckel N, Li Y, Kuxhaus O, Stefan N, Hu FB, Schulze MB. Transition from metabolic healthy to unhealthy phenotypes and association with cardiovascular disease risk across BMI categories in 90 257 women (the Nurses’ Health Study): 30 year follow-up from a prospective cohort study. Lancet Diabetes Endocrinol. 2018;6(9):714–24. 10.1016/S2213-8587(18)30137-2. 29859908 10.1016/S2213-8587(18)30137-2

[36] Krolick KN, Shi H. Estrogenic action in stress-induced neuroendocrine regulation of energy homeostasis. Cells. 2022. 10.3390/cells11050879. 35269500 10.3390/cells11050879 PMC8909319

[37] Lee C, Tsenkova VK, Boylan JM, Ryff CD. Gender differences in the pathways from childhood disadvantage to metabolic syndrome in adulthood: an examination of health lifestyles. SSM - Population Health. 2018;4:216–24. 10.1016/j.ssmph.2018.01.003. 29854905 10.1016/j.ssmph.2018.01.003 PMC5976858

[38] Wright L, Bukowski WM. Gender is key: girls’ and boys’ cortisol differs as a factor of socioeconomic status and social experiences during early adolescence. J Youth Adolesc. 2021;50(6):1281–91. 10.1007/s10964-020-01382-z. 33515375 10.1007/s10964-020-01382-z

[39] Xi B, Zong Xn, Kelishadi R, et al. International waist circumference percentile cutoffs for central obesity in children and adolescents aged 6 to 18 years. J Clin Endocrinol Metab. 2020;105(4):e1569–83. 10.1210/clinem/dgz195. 31723976 10.1210/clinem/dgz195 PMC7059990

