# Supplementary material for: Sex differences in metabolic syndrome among U.S. adolescents, NHANES 1999–2020
Source: Diabetol Metab Syndr. 2025 Dec 11;18:19. doi: 10.1186/s13098-025-02052-5 (PMC12801445; doi:10.1186/s13098-025-02052-5)
Supplement: Supplementary file 1 — Supplementary Material 1 [file 13098_2025_2052_MOESM1_ESM.docx]

**Supplemental Materials**

**[Supplemental methods.](#_Toc32420)** [Definition of Metabolic Syndrome and Metabolically Healthy/Unhealthy Obesity 2](#_Toc32420)

**[Table S1.](#_Toc25293)** [Sex Differences in the Five Metabolic Indices among Adolescents, Stratified by Age Groups 5](#_Toc25293)

**[Table S2.](#_Toc16902)** [Sex Differences in the Five Metabolic Indices under Multiple Definition Standards, Stratified by BMI Categories 7](#_Toc16902)

**[Table S3.](#_Toc3380)** [Prevalence and Sex Disparities of Metabolic Syndrome in Adolescents across Different Definition Criteria 9](#_Toc3380)

**[Table S4.](#_Toc3405)** [Sex Differences in the Metabolic Phenotype among Adolescents 11](#_Toc3405)

**[Table S5.](#_Toc21383)** [Prevalence and Sex Disparities of Metabolic Syndrome in Adolescents When AO was Defined as WHtR≥0.5 12](#_Toc21383)

**[Table S6.](#_Toc11006)** [Criteria and Prevalence of Metabolic Syndrome among Adolescents in Previous Studies 13](#_Toc11006)

**[Figure S1.](#_Toc27929)** [Study Flowchart 17](#_Toc27929)

**[Figure S2.](#_Toc14452)** [Temporal Trends of Metabolic Indices among Adolescents, Overall and by Sex 18](#_Toc14452)

**[Figure S3.](#_Toc8141)** [Metabolic Risk Factor Combinations in Adolescents with Metabolic Syndrome, Overall and by Sex 19](#_Toc8141)

**[Figure S4.](#_Toc21044)** [Trends in Metabolic Syndrome Prevalence (Defined by Alternative Criteria) in Adolescents: By Survey Cycle and Age 20](#_Toc21044)

**[Figure S5.](#_Toc29589)** [Prevalence of Metabolic Syndrome (Defined by Alternative Criteria) in Adolescents by Subgroups 21](#_Toc29589)

**[References for supplemental materials](#_Toc20452)** [22](#_Toc20452)

# **Supplemental methods. Definition of Metabolic Syndrome and Metabolically Healthy/Unhealthy Obesity**

Various diagnostic criteria have been proposed by different organizations to define metabolic syndrome (MS) over the past decade. The MS is commonly recognized as consisting of five risk factors, namely central obesity, raised blood pressure (BP), elevated fasting glucose (FPG), and dyslipidemia (characterized by increased triglyceride [TG] and lowered high-density lipoprotein cholesterol [HDL-C]) .

To date, there has yet to be a unified definition that can be employed to evaluate the risk among children and adolescents. We applied two classification systems to examine the presence of **MS**. The first system adopted the latest joint scientific statement on MS,^1^ which declared that three abnormal findings out of five risk factors would qualify a person for MS. The other system used remained taking the International Diabetes Federation (IDF) definition with central obesity being an obligatory component.^2^ The criteria for risk factors are listed in the table below:

|  | **Criterion 1** | **Criterion 2** | | **Criterion 3** |
| --- | --- | --- | --- | --- |
| **Waist circumference (WC)** | <16 years: ≥90th percentile (age and sex specific) or adult cutoff if lower ≥16 years: >102 cm in males and>88 cm in females | | | |
| **Blood pressure (BP)** | ≥130/85 mmHg | ≥90th percentile (age, sex, and height specific) | | |
| **Fasting plasma glucose (FPG)** | ≥100 mg/dL (5.6 mmol/L) | | | |
| **Triglycerides (TG)** | ≥150 mg/dL (1.7 mmol/L) | | | ≥110 mg/dL (1.24 mmol/L) |
| **High-density lipoprotein cholesterol (HDL-C)** | <16 years:<40 mg/dL (1.03 mmol/L)  ≥16 years:<40 mg/dL (1.03 mmol/L) in males and <50 mg/dL (1.29 mmol/L) in females | | | ≤40 mg/dL (1.03 mmol/L) |
| **Reference** | *Zimmet et al. 2007a; Liu et al. 2022;*  *Liu et al. 2023* | | *Zimmet et al. 2007b; Cai et al. 2022* | *Cook et al. 2003; Duncan, Li, and Zhou 2004* |

Regarding each risk factor, since the criteria in the literature are not completely unified, there are different cutoff points for defining abnormal values. **Criterion 1** was the primary one adopted in our article. Besides waist circumference (WC), it also included BP≥130/85 mmHg, FPG≥100 mg/dL, TG≥150 mg/dL, and HDL-C<40 mg/dL (for males and females under 16 years old) or <50 mg/dL (for females aged 16 years and above). The definition for these risk factors have been widely used in many studies.^3-5^ **Criterion 2** differed from Criterion 1 in that the criterion for abnormally increased BP adopted ≥90th percentile by age, sex, and height, which is still often applied in articles related to adolescent metabolism-related studies.^6,7^ **Criterion 3** was more frequently used in earlier articles and mainly further adjusted the criterion for dyslipidemia in adolescents on the basis of Criterion 2.^8,9^ However, its application has been relatively rare in recent years. Moreover, most of these articles identified FPG≥110 mg/dL (≥6.1 mmol/L) as elevated, referring to the definition of the National Cholesterol Education Program (NCEP) Adult Treatment Panel III (ATP III) (2001), and this standard was modified in 2004 to≥100 mg/dL (5.6 mmol/L) in 2004. Therefore, this cutoff of FPG was not considered in this study.

To define metabolically healthy obesity (MHO) and metabolically unhealthy obesity (MUO), our study first used the percentile values of body mass index (BMI) to identify obesity among adolescents. The age- and sex-specific BMI percentiles were computed by employing reference data from the Centers for Disease Control and Prevention and were categorized into three groups: underweight (<5th percentile)/normal weight (5th to <85th percentile), overweight (85th to 95th percentile), and obese (≥95th percentile).^5^ WC was excluded due to its collinearity with BMI. Similar with some previous research,^7,10-12^ adolescents with obesity were further classified as having **MHO** if they had none of the four metabolic components or as having **MUO** if they had one to four metabolic abnormalities (specifically, high BP, elevated FPG, increased TG, or low HDL-C). The definitions of the four metabolic indicator abnormalities also reference the criteria presented in the above table. Among these, the results following Criterion 1 are primarily described in the main text. Moreover, we also repeated the analyses including overweight individuals. Besides, we also noticed that some articles directly applied the definition of MS to define "metabolic health status" when defining MHO/MUO, determining it on the basis of the presence or absence of MS (from WC, BP, FPG, TG and HDL-C) and obesity (from BMI).^5,9^ This approach was not used in our article.

# **Table S1. Sex Differences in the Five Metabolic Indices among Adolescents, Stratified by Age Groups**

|  | **Age of 12-15 years (n=3458)** | | |  | **Age of 16-19 years (n=3531)** | | |
| --- | --- | --- | --- | --- | --- | --- | --- |
|  | **Male (n=1783)** | **Female (n=1675)** | **P value** |  | **Male (n=1861)** | **Female (n=1670)** | **P value** |
| **Waist circumference** |  |  |  |  |  |  |  |
| STD1: <16 years: ≥90th percentile or adult cutoff if lower; ≥16 years: >102 cm in males and >88 cm in females | 10.49 (8.35, 12.63) | 22.14 (18.88, 25.4)^a^ | <0.001 |  | 14.77 (12.06, 17.48)^b^ | 29.58 (26.29, 32.86)^c^ | <0.001 |
| STD2: ≥102 cm in males and ≥88 cm in females | 9.04 (7.1, 10.98) | 22.14 (18.88, 25.4) | <0.001 |  | 14.77 (12.06, 17.48)^b^ | 29.58 (26.29, 32.86)^c^ | <0.001 |
| STD3: ≥94 cm in males and ≥80 cm in females | 14.3 (11.83, 16.77) | 37.5 (33.95, 41.06) | <0.001 |  | 24.6 (21.11, 28.09)^b^ | 49.19 (45.78, 52.59)^c^ | <0.001 |
| **Blood pressure** |  |  |  |  |  |  |  |
| STD1: ≥130/85 mmHg | 3.25 (1.85, 4.66) | 0.3 (0.02, 0.58) | <0.001 |  | 8.56 (6.4, 10.73)^b^ | 1.52 (0.7, 2.33)^c^ | <0.001 |
| STD2: ≤17 years: ≥90th percentile by age, sex, and height; >17 years: ≥130/85 mmHg | 9.75 (7.74, 11.76) | 8.1 (6, 10.21) | 0.281 |  | 10.65 (8.4, 12.89) | 5.49 (4.07, 6.92)^c^ | <0.001 |
| **Fasting plasma glucose** |  |  |  |  |  |  |  |
| STD1: ≥100 mg/dL | 23.92 (20.57, 27.26) | 12.05 (10.06, 14.03) | <0.001 |  | 21.57 (18.5, 24.64) | 8.78 (6.6, 10.95)^c^ | <0.001 |
| STD2: ≥110 mg/dL | 2.22 (1.3, 3.14) | 1.66 (0.76, 2.56) | 0.406 |  | 3.36 (2.32, 4.4) | 1.61 (0.76, 2.45) | 0.020 |
| **Triglycerides** |  |  |  |  |  |  |  |
| STD1: ≥150 mg/dL | 7.66 (5.7, 9.62) | 5.82 (4.21, 7.42) | 0.158 |  | 10.03 (8.14, 11.93) | 6.95 (5.25, 8.65) | 0.023 |
| STD2: ≥110 mg/dL | 17.53 (14.97, 20.08) | 15.8 (13.09, 18.5) | 0.360 |  | 22.06 (19.14, 24.99)^b^ | 16.97 (14.48, 19.46) | 0.005 |
| **High-density lipoprotein cholesterol** |  |  |  |  |  |  |  |
| STD1: <16 years: <40 mg/dL; ≥16 years: <40 mg/dL in males and <50 mg/dL in females | 15.34 (12.93, 17.76) | 9.13 (7.24, 11.02) | <0.001 |  | 22.19 (19.58, 24.79)^b^ | 36.41 (33.16, 39.66)^c^ | <0.001 |
| STD2: ≤40 mg/dL | 17.49 (14.91, 20.07) | 11.47 (9.21, 13.73) | 0.001 |  | 25.67 (22.88, 28.47)^b^ | 11.55 (9.36, 13.74) | <0.001 |

Data are presented as percentage (95%CI), adjusted for NHANES fasting sample weight.

CI=confidence interval; NHANES=National Health and Nutrition Examination Survey; STD=standard.

^a^ According to our calculations, the age- and sex-specific 90th percentiles of waist circumference for females under 16 years old actually exceeded the adult cutoff value (88 cm). Therefore, 88 cm was actually adopted.

^b^ There was a statistically significant difference in the proportion of abnormalities between the 12-15-year-old and 16-19-year-old age groups among males.

^c^ There was a statistically significant difference in the proportion of abnormalities between the 12-15-year-old and 16-19-year-old age groups among females.

# **Table S2. Sex Differences in the Five Metabolic Indices under Multiple Definition Standards, Stratified by BMI Categories**

|  | **Underweight/normal weight (n=4343)** | | |  | **Overweight (n=1200)** | | |  | **Obesity (n=1446)** | | |
| --- | --- | --- | --- | --- | --- | --- | --- | --- | --- | --- | --- |
|  | **Male (n=2323)** | **Female (n=2020)** | **P value** |  | **Male (n=575)** | **Female (n=625)** | **P value** |  | **Male (n=746)** | **Female (n=700)** | **P value** |
| **Waist circumference** |  |  |  |  |  |  |  |  |  |  |  |
| STD1: <16 years:≥90th percentile or adult cutoff if lower; ≥16 years: >102 cm in males and >88 cm in females | 0.00 | 2.08 | <0.001 |  | 2.47 | 40.01 | <0.001 |  | 61.62 | 91.38 | <0.001 |
| STD2: ≥102 cm in males and ≥88 cm in females | 0.00 | 2.08 | <0.001 |  | 2.15 | 40.01 | <0.001 |  | 58.25 | 91.38 | <0.001 |
| STD3: ≥94 cm in males and ≥80 cm in females | 0.47 | 15.87 | <0.001 |  | 17.08 | 82.38 | <0.001 |  | 83.12 | 99.78 | <0.001 |
| **Blood pressure** |  |  |  |  |  |  |  |  |  |  |  |
| STD1: ≥130/85 mmHg | 3.09 | 0.34 | <0.001 |  | 6.84 | 0.67 | <0.001 |  | 14.40 | 2.96 | <0.001 |
| STD2: ≤17 years: ≥90th percentile by age, sex, and height; >17 years: ≥130/85 mmHg | 5.86 | 4.65 | 0.215 |  | 14.39 | 8.67 | 0.041 |  | 21.08 | 12.20 | 0.001 |
| **Fasting plasma glucose** |  |  |  |  |  |  |  |  |  |  |  |
| STD1: ≥100 mg/dL | 20.16 | 8.60 | <0.001 |  | 21.49 | 9.81 | <0.001 |  | 32.10 | 16.81 | <0.001 |
| STD2: ≥110 mg/dL | 2.20 | 1.12 | 0.067 |  | 3.54 | 1.43 | 0.046 |  | 4.15 | 3.47 | 0.612 |
| **Triglycerides** |  |  |  |  |  |  |  |  |  |  |  |
| STD1: ≥150 mg/dL | 3.95 | 4.31 | 0.694 |  | 11.18 | 6.79 | 0.048 |  | 22.98 | 12.82 | 0.001 |
| STD2: ≥110 mg/dL | 13.25 | 12.18 | 0.456 |  | 21.18 | 18.96 | 0.465 |  | 40.08 | 27.91 | 0.002 |
| **High-density lipoprotein cholesterol** |  |  |  |  |  |  |  |  |  |  |  |
| STD1: <16 years: <40 mg/dL; ≥16 years: <40 mg/dL in males and <50 mg/dL in females | 11.57 | 16.70 | 0.002 |  | 19.68 | 28.17 | 0.031 |  | 41.51 | 38.16 | 0.388 |
| STD2: ≤40 mg/dL | 13.61 | 7.17 | <0.001 |  | 22.05 | 13.15 | 0.007 |  | 47.23 | 24.25 | <0.001 |

Data are presented as percentage (%), adjusted for NHANES fasting sample weight.

BMI=body mass index; NHANES=National Health and Nutrition Examination Survey; STD=standard.

# **Table S3. Prevalence and Sex Disparities of Metabolic Syndrome in Adolescents across Different Definition Criteria**

|  | **Overall (n=6989)** | **Male (n=3644)** | **Female (n=3345)** | **P value** |  |
| --- | --- | --- | --- | --- | --- |
| **MS in classification system 1: defined as ≥3 of 5 risk factors** | | | | | |
| **Criterion 1** | **5.13 (4.31, 5.94)** | **6.11 (4.86, 7.37)** | **4.07 (3.02, 5.11)** | 0.017 |  |
| number of other risk factors |  |  |  | 0.005 |  |
| 3 | 4.11 (3.37, 4.85) | 4.84 (3.68, 5.99) | 3.33 (2.44, 4.22) |  |  |
| 4 | 0.8 (0.48, 1.12) | 0.86 (0.43, 1.29) | 0.73 (0.26, 1.2) |  |  |
| 5 | 0.22 (0.08, 0.36) | 0.42 (0.15, 0.68) | 0.01 (-0.01, 0.03) |  |  |
| **Criterion 2** | **5.72 (4.86, 6.59)** | **6.54 (5.25, 7.83)** | **4.85 (3.73, 5.97)** | 0.055 |  |
| number of other risk factors |  |  |  | 0.039 |  |
| 3 | 4.44 (3.68, 5.21) | 5.05 (3.91, 6.19) | 3.79 (2.82, 4.77) |  |  |
| 4 | 0.99 (0.67, 1.31) | 1.05 (0.58, 1.51) | 0.92 (0.47, 1.38) |  |  |
| 5 | 0.29 (0.12, 0.47) | 0.44 (0.17, 0.71) | 0.13 (-0.02, 0.29) |  |  |
| **Criterion 3** | **7.33 (6.35, 8.3)** | **8.98 (7.51, 10.45)** | **5.56 (4.39, 6.72)** | <0.001 |  |
| number of other risk factors |  |  |  | <0.001 |  |
| 3 | 5.43 (4.69, 6.17) | 6.52 (5.4, 7.64) | 4.27 (3.3, 5.24) |  |  |
| 4 | 1.5 (1.09, 1.91) | 1.91 (1.27, 2.55) | 1.06 (0.61, 1.51) |  |  |
| 5 | 0.4 (0.16, 0.63) | 0.55 (0.25, 0.85) | 0.23 (-0.04, 0.5) |  |  |
| **MS in classification system 2: defined as AO and ≥2 of 4 risk factors** | | | | | |
| **Criterion 1** | **4.49 (3.7, 5.28)** | **5.08 (3.91, 6.25)** | **3.86 (2.82, 4.9)** | 0.127 |  |
| number of other risk factors |  |  |  | <0.001 |  |
| 2 | 3.49 (2.79, 4.18) | 3.83 (2.78, 4.87) | 3.12 (2.25, 3.99) |  |  |
| 3 | 0.78 (0.46, 1.1) | 0.83 (0.4, 1.26) | 0.73 (0.26, 1.2) |  |  |
| 4 | 0.22 (0.08, 0.36) | 0.42 (0.15, 0.68) | 0.01 (-0.01, 0.03) |  |  |
| **Criterion 2** | **4.94 (4.11, 5.77)** | **5.34 (4.14, 6.53)** | **4.52 (3.42, 5.62)** | 0.319 |  |
| number of other risk factors |  |  |  | <0.001 |  |
| 2 | 3.71 (3.02, 4.4) | 3.88 (2.86, 4.89) | 3.53 (2.6, 4.45) |  |  |
| 3 | 0.94 (0.61, 1.26) | 1.01 (0.56, 1.47) | 0.86 (0.39, 1.33) |  |  |
| 4 | 0.29 (0.12, 0.47) | 0.44 (0.17, 0.71) | 0.13 (-0.02, 0.29) |  |  |
| **Criterion 3** | **5.93 (5.04, 6.82)** | **6.6 (5.35, 7.85)** | **5.21 (4.04, 6.39)** | 0.106 |  |
| number of other risk factors |  |  |  | <0.001 |  |
| 2 | 4.08 (3.45, 4.72) | 4.17 (3.27, 5.07) | 3.99 (3.04, 4.94) |  |  |
| 3 | 1.45 (1.03, 1.87) | 1.88 (1.24, 2.52) | 0.99 (0.53, 1.46) |  |  |
| 4 | 0.4 (0.16, 0.63) | 0.55 (0.25, 0.85) | 0.23 (-0.04, 0.5) |  |  |

Data are presented as percentage (95%CI), adjusted for NHANES fasting sample weight.

AO=abdominal obesity; NHANES=National Health and Nutrition Examination Survey; CI=confidence interval.

# **Table S4. Sex Differences in the Metabolic Phenotype among Adolescents**

|  | **Overall (n=6898)** | **Male (n=3644)** | **Female (n=3345)** | **P value** |
| --- | --- | --- | --- | --- |
| **Metabolically healthy obesity** |  |  |  |  |
| Criterion 1 | 7.84% (6.93, 8.74) | 6.22% (5.04, 7.4) | 9.57% (7.96, 11.18) | 0.002 |
| Criterion 2 | 7.17% (6.28, 8.06) | 5.67% (4.53, 6.81) | 8.77% (7.19, 10.36) | 0.004 |
| Criterion 3 | 6.84% (5.93, 7.74) | 4.5% (3.41, 5.59) | 9.34% (7.82, 10.85) | <0.001 |
| **Metabolically unhealthy obesity** |  |  |  |  |
| Criterion 1 | 11.9% (10.77, 13.03) | 13.67% (11.85, 15.49) | 10.01% (8.64, 11.38) | 0.002 |
| Criterion 2 | 12.57% (11.41, 13.72) | 14.22% (12.38, 16.06) | 10.8% (9.38, 12.23) | 0.005 |
| Criterion 3 | 12.9% (11.69, 14.11) | 15.39% (13.45, 17.34) | 10.24% (8.7, 11.78) | <0.001 |
| **Metabolically healthy overweight/obesity** |  |  |  |  |
| Criterion 1 | 17.05% (15.82, 18.28) | 14.67% (12.88, 16.45) | 19.6% (17.58, 21.62) | 0.001 |
| Criterion 2 | 15.55% (14.39, 16.72) | 13.34% (11.65, 15.02) | 17.92% (15.95, 19.9) | 0.002 |
| Criterion 3 | 15.4% (14.2, 16.61) | 11.49% (9.82, 13.16) | 19.58% (17.64, 21.52) | <0.001 |
| **Metabolically unhealthy overweight/obesity** |  |  |  |  |
| Criterion 1 | 18.68% (17.29, 20.07) | 20.61% (18.53, 22.7) | 16.61% (14.85, 18.38) | 0.004 |
| Criterion 2 | 20.18% (18.75, 21.6) | 21.94% (19.85, 24.04) | 18.29% (16.41, 20.16) | 0.011 |
| Criterion 3 | 20.33% (18.87, 21.79) | 23.79% (21.6, 25.98) | 16.63% (14.78, 18.48) | <0.001 |

Data are presented as percentage (95%CI), adjusted for NHANES fasting sample weight.

NHANES=National Health and Nutrition Examination Survey.

# **Table S5. Prevalence and Sex Disparities of Metabolic Syndrome in Adolescents When AO was Defined as WHtR≥0.5**

|  | **Overall (n=6989)** | **Male (n=3644)** | **Female (n=3345)** | **P value** |
| --- | --- | --- | --- | --- |
| **MS in classification system 1: defined as ≥3 of 5 risk factors** | | | | |
| **Criterion 1** | **6.48 (5.55, 7.4)** | **8.18 (6.71, 9.64)** | **4.66 (3.56, 5.76)** | <0.001 |
| number of other risk factors |  |  |  | <0.001 |
| 3 | 5.14 (4.3, 5.97) | 6.34 (5.01, 7.67) | 3.85 (2.92, 4.79) |  |
| 4 | 1.1 (0.74, 1.46) | 1.38 (0.86, 1.91) | 0.8 (0.31, 1.29) |  |
| 5 | 0.24 (0.1, 0.37) | 0.45 (0.18, 0.72) | 0.01 (-0.01, 0.03) |  |
| **Criterion 2** | **7.62 (6.63, 8.61)** | **9.12 (7.58, 10.66)** | **6.03 (4.81, 7.24)** | 0.002 |
| number of other risk factors |  |  |  | 0.003 |
| 3 | 6 (5.13, 6.87) | 7.03 (5.67, 8.38) | 4.9 (3.85, 5.94) |  |
| 4 | 1.32 (0.95, 1.68) | 1.62 (1.06, 2.18) | 1 (0.52, 1.47) |  |
| 5 | 0.31 (0.13, 0.49) | 0.47 (0.2, 0.75) | 0.13 (-0.02, 0.29) |  |
| **Criterion 3** | **9.97 (8.77, 11.16)** | **12.77 (10.94, 14.6)** | **6.97 (5.65, 8.3)** | <0.001 |
| number of other risk factors |  |  |  | <0.001 |
| 3 | 7.4 (6.46, 8.34) | 9.11 (7.71, 10.51) | 5.57 (4.43, 6.71) |  |
| 4 | 2.16 (1.68, 2.63) | 3.08 (2.3, 3.86) | 1.17 (0.7, 1.64) |  |
| 5 | 0.41 (0.17, 0.65) | 0.58 (0.28, 0.89) | 0.23 (-0.04, 0.5) |  |
| **MS in classification system 2: defined as AO and ≥2 of 4 risk factors** | | | | |
| **Criterion 1** | **6.18 (5.27, 7.09)** | **7.72 (6.33, 9.12)** | **4.52 (3.42, 5.63)** | 0.001 |
| **Criterion 2** | **7.2 (6.24, 8.17)** | **8.55 (7.08, 10.01)** | **5.77 (4.57, 6.96)** | 0.004 |
| **Criterion 3** | **9.26 (8.13, 10.39)** | **11.62 (9.96, 13.29)** | **6.74 (5.4, 8.09)** | <0.001 |

Data are presented as percentage (95%CI), adjusted for NHANES fasting sample weight.

AO=abdominal obesity; NHANES=National Health and Nutrition Examination Survey; CI=confidence interval.

# **Table S6. Criteria and Prevalence of Metabolic Syndrome among Adolescents in Previous Studies**

| **Variables** | **Cook et al. 2003^8^** | **de Ferranti et al. 2004^13^** | **Weiss et al. 2004^14^** | **Cruz et al. 2004^15^** | **Duncan, Li, and Zhou 2004^9^** | **Ford, Ajani, and Mokdad 2005^16^** | **Kranz, Mahood, and Wagstaff 2007^17^** | **Ahrens et al. 2014^18^: monitoring level (action level)** | **Liu et al. 2022^4^, 2023^5^** |
| --- | --- | --- | --- | --- | --- | --- | --- | --- | --- |
| **Study population** | 12-19 years, NHANES 1988-1994 | 12-19 years, NHANES 1988-1994 | Obese children and adolescents between 4 and 20 years of age | Overweight children aged 8-13years with a family history for type 2 diabetes | 12-19 years, NHANES 1999-2000 | 12-17 years, NHANES 1999-2000 | Children 2-18 years old of a nationally representative data set (NHANES 1999–2002), with subgroups of 12-18 years | Population-based survey in eight European countries, including children 2.0-10.9 year | 12-19 years, NHANES 1999-2018 |
| **Requirement of MS** | ≥3 criteria | ≥3 criteria | ≥3 criteria | ≥3 criteria | ≥3 criteria | ≥3 criteria | ≥3 criteria (Cook) | ≥3 criteria | central obesity and presence of 2 or more other risk factors |
| **WC** | ≥90th percentile | ≥75th percentile | – | ≥90th percentile | ≥90th percentile | ≥90th percentile | ≥90th percentile | ≥90th (95th) percentile | ≥90th percentile for 10 to <16 years;  >102 cm in men and >88 cm in women for 16 years |
| **SBP** | ≥90th percentile | ≥90th percentile | ≥95th percentile | ≥90th percentile | ≥90th percentile | ≥90th percentile | ≥90th percentile | ≥90th (95th) percentile | ≥130 mmHg |
| **DBP** | ≥90th percentile | ≥90th percentile | ≥95th percentile | ≥90th percentile | ≥90th percentile | ≥90th percentile | ≥90th percentile | ≥90th (95th) percentile | ≥85 mmHg |
| **TG** | ≥1.24 mmol/L | ≥1.1 mmol/L | ≥95th percentile | ≥90th percentile | ≥110 mg/dl | ≥110 mg/dl | ≥110 mg/dl | ≥90th (95th) percentile | ≥150 mg/dL |
| **HDL-C** | ≤1.03 mmol/L | ≤1.3 mmol/L ;  ≤1.17 mmol/L for boys aged 15–19 years | ≤5th percentile | ≤10th percentile | ≤40 mg/dl | ≤40 mg/dl | ≤40 mg/dl | ≤10th (5th) percentile | <40 mg/dL for 10 to <16 years;  <40 mg/dL in men and <50 mg/dL in women for16 years |
| **Glucose** | FG≥6.11 mmol/L | FG≥6.1 mmol/L | GI (ADA criteria) | impaired glucose tolerance | ≥110 mg/dl | ≥110 mg/dl | ≥100 mg/dl | FG or HOMA-IR>90th (95th) percentile | ≥100 mg/dL |
| **BMI** | – | – | z-score≥2 |  | – |  |  | – | – |
| **Prevalence of MS according to study** | Overall prevalence was 4.2%; 6.1% of males and 2.1% of females were affected (P=0.01). | 9.2% qualified as having MS; Prevalence was comparable for boys and girls (9.5% vs 8.9% ) and for older and younger adolescents (8.3% vs 10.3%). | The overall prevalence of the MS was 38.7% in moderately obese subjects and 49.7% in severely obese subjects. | 30.2% of overweight Hispanic subjects with three or more components of the MS. | Overall prevalence was 6.4%;  MS was more common in male (9.1%) than in female (3.7%) adolescents (P<0.01). | The MS prevalence was 5.2%; 6.3% among males and 4.1% among females (P = 0.233). | Subgroups aged 12-18 years who were not overweight or obese: 0.7%;  those who were classified to be overweight or obese: 23%. | Among the various definitions, the highest prevalence (5.5%) was obtained with proposed new definition requiring close observation (monitoring level); While more conservative definition, requiring intervention gives a prevalence of 1.8%. | The prevalence of MS remained stable at 4.36% (female 3.44%, male 5.53%), whereas prevalence trends of subcomponents were varied. |

BMI=body mass index; DBP=diastolic blood pressure; FG=fasting glucose; GI=glucose intolerance; HDL-C=high-density lipoprotein cholesterol; HOMA-IR=Homeostatic Model Assessment of Insulin Resistance; MS=metabolic syndrome; NHANES=National Health and Nutrition Examination Survey; SBP=systolic blood pressure; TG=triglycerides; WC=waist circumference.

# **Figure S1. Study Flowchart**

BMI=body mass index; BP=blood pressure; DBP=diastolic blood pressure; FPG=fasting plasma glucose; HDL-C=high-density lipoprotein cholesterol; NHANES=National Health and Nutrition Examination Survey; SBP=systolic blood pressure; TG=triglycerides; WC=waist circumference.

^a^ A subset of participants were randomly selected to attend the morning session after an overnight fast in the National Health and Nutrition Examination Survey (NHANES).

^b^ Multiple exclusions may apply to one participant, so the sum of the numbers in each criterion may be greater than the total number excluded.

^c^ There were at most four measurements for SBP and DBP. This study obtained the average BP values by averaging all the recorded values. A total of 366 people had no measured values for either SBP or DBP.

# **Figure S2. Temporal Trends of Metabolic Indices among Adolescents, Overall and by Sex**

BP=blood pressure; FPG=fasting plasma glucose; HDL-C=high-density lipoprotein cholesterol; MS=metabolic syndrome; NHANES=National Health and Nutrition Examination Survey; TG=triglycerides; WC=waist circumference.

# **Figure S3. Metabolic Risk Factor Combinations in Adolescents with Metabolic Syndrome, Overall and by Sex**

The bar chart on the left shows the proportions of metabolic risk factor combinations for all populations (black), males (blue), and females (red). The table on the right presents specific values by percentage (95% CI), adjusted for NHANES fasting sample weight. Additionally, the top five combinations are highlighted in bold.

AO=abdominal obesity; BP=blood pressure; FPG=fasting plasma glucose; HDL-C=high-density lipoprotein cholesterol; TG=triglycerides.

# **Figure S4. Trends in Metabolic Syndrome Prevalence (Defined by Alternative Criteria) in Adolescents: By Survey Cycle and Age**

MS=metabolic syndrome.

# **Figure S5. Prevalence of Metabolic Syndrome (Defined by Alternative Criteria) in Adolescents by Subgroups**

BMI=body mass index; MS=metabolic syndrome; PIR=family poverty income ratio.

# **References for supplemental materials**

1. Alberti KGMM, Eckel RH, Grundy SM, et al. Harmonizing the metabolic syndrome: a joint interim statement of the International Diabetes Federation Task Force on Epidemiology and Prevention; National Heart, Lung, and Blood Institute; American Heart Association; World Heart Federation; International Atherosclerosis Society; and International Association for the Study of Obesity. *Circulation*. 2009;120(16):1640-1645. doi:10.1161/CIRCULATIONAHA.109.192644

2. Alberti KGMM, Zimmet P, Shaw J. The metabolic syndrome—a new worldwide definition. *Lancet*. 2005;366(9491):1059-1062.

3. Zimmet P, Alberti G, Kaufman F, et al. The metabolic syndrome in children and adolescents. *Lancet*. 2007;369(9579):2059-2061.

4. Liu J, Ma J, Orekoya O, Vangeepuram N, Liu J. Trends in Metabolic Syndrome Among US Youth, From 1999 to 2018. *JAMA Pediatr*. 2022;176(10):1043-1045. doi:10.1001/jamapediatrics.2022.1850

5. Liu J, Park Y-MM, Ma J, Lavie CJ. Trends in Metabolic Phenotypes of Obesity Among US Adolescents, NHANES 1999-2018. *Mayo Clin Proc*. 2023;98(4):633-636. doi:10.1016/j.mayocp.2023.01.025

6. Zimmet P, Alberti KGM, Kaufman F, et al. The metabolic syndrome in children and adolescents - an IDF consensus report. *Pediatr Diabetes*. 2007;8(5):299-306.

7. Cai S, Dang J, Zhong P, et al. Sex differences in metabolically healthy and metabolically unhealthy obesity among Chinese children and adolescents. *Front Endocrinol (Lausanne)*. 2022;13:980332. doi:10.3389/fendo.2022.980332

8. Cook S, Weitzman M, Auinger P, Nguyen M, Dietz WH. Prevalence of a metabolic syndrome phenotype in adolescents: findings from the third National Health and Nutrition Examination Survey, 1988-1994. *Arch Pediatr Adolesc Med*. 2003;157(8):821-827.

9. Duncan GE, Li SM, Zhou X-H. Prevalence and trends of a metabolic syndrome phenotype among U.S. adolescents, 1999-2000. *Diabetes Care*. 2004;27(10):2438-2443.

10. Zhao M, López-Bermejo A, Caserta CA, et al. Metabolically Healthy Obesity and High Carotid Intima-Media Thickness in Children and Adolescents: International Childhood Vascular Structure Evaluation Consortium. *Diabetes Care*. 2019;42(1):119-125. doi:10.2337/dc18-1536

11. Prince RL, Kuk JL, Ambler KA, Dhaliwal J, Ball GDC. Predictors of metabolically healthy obesity in children. *Diabetes Care*. 2014;37(5):1462-1468. doi:10.2337/dc13-1697

12. Wang J-S, Xia P-F, Ma M-N, et al. Trends in the Prevalence of Metabolically Healthy Obesity Among US Adults, 1999-2018. *JAMA Netw Open*. 2023;6(3):e232145. doi:10.1001/jamanetworkopen.2023.2145

13. de Ferranti SD, Gauvreau K, Ludwig DS, Neufeld EJ, Newburger JW, Rifai N. Prevalence of the metabolic syndrome in American adolescents: findings from the Third National Health and Nutrition Examination Survey. *Circulation*. 2004;110(16):2494-2497.

14. Weiss R, Dziura J, Burgert TS, et al. Obesity and the metabolic syndrome in children and adolescents. *N Engl J Med*. 2004;350(23):2362-2374.

15. Cruz ML, Weigensberg MJ, Huang TTK, Ball G, Shaibi GQ, Goran MI. The metabolic syndrome in overweight Hispanic youth and the role of insulin sensitivity. *J Clin Endocrinol Metab*. 2004;89(1):108-113.

16. Ford ES, Ajani UA, Mokdad AH. The metabolic syndrome and concentrations of C-reactive protein among U.S. youth. *Diabetes Care*. 2005;28(4):878-881.

17. Kranz S, Mahood LJ, Wagstaff DA. Diagnostic criteria patterns of U.S. children with Metabolic Syndrome: NHANES 1999-2002. *Nutr J*. 2007;6:38.

18. Ahrens W, Moreno LA, Mårild S, et al. Metabolic syndrome in young children: definitions and results of the IDEFICS study. *Int J Obes (Lond)*. 2014;38 Suppl 2:S4-14. doi:10.1038/ijo.2014.130
